# Congenital exercise ability ameliorates muscle atrophy but not spinal cord recovery in spinal cord injury mouse model

**DOI:** 10.7150/ijms.37442

**Published:** 2019-10-21

**Authors:** Po-An Tai, Yi-Ju Hsu, Wen-Ching Huang, Chun-Hao Chang, Yi-Hsun Chen, Chi-Chang Huang, Li Wei

**Affiliations:** 1 Division of Neurosurgery, Department of Surgery, Taipei Tzu Chi Hospital, Buddhist Tzu Chi Medical Foundation, New Taipei City 23142, Taiwan.; 2 Department of Surgery, School of Medicine, Buddhist Tzu Chi University, Hualien County 97004, Taiwan; 3 Graduate Institute of Sports Science, National Taiwan Sport University, Taoyuan 33301, Taiwan;; 4 Department of Exercise and Health Science, National Taipei University of Nursing and Health Sciences, Taipei 11219, Taiwan;; 5 Graduate Institute of Veterinary Pathobiology, National Chung Hsin University, Taichung, 402, Taiwan;;; 6 Graduate Institute of Injury Prevention and Control, College of Public Health, Taipei Medical University, Taipei 11031, Taiwan.

**Keywords:** spinal cord injury, congenital exercise ability, muscle atrophy

## Abstract

Spinal cord injury (SCI) can cause loss of mobility in the limbs, and no drugs, surgical procedures, or rehabilitation strategies provide a complete cure. Exercise capacity is thought to be associated with the causes of many diseases. However, no studies to date have assessed whether congenital exercise ability is related to the recovery of spinal cord injury. High congenital exercise ability (HE) and low congenital exercise ability (LE) mice were artificially bred from the same founder ICR mice. The HE and LE groups still exhibited differences in exercise ability after 13 generations of breeding. Histological staining and immunohistochemistry staining indicated no significant differences between the HE and LE groups on recovery of the spinal cord. In contrast, after SCI, the HE group exhibited better mobility in gait analysis and longer endurance times in the exhaustive swimming test than the LE group. In addition, after SCI, the HE group also exhibited less atrophy than the LE group, and no inflammatory cells appeared. In conclusion, we found that high congenital exercise ability may reduce the rate of muscle atrophy. This result can be applied to sports science and rehabilitation science as a reference for preventive medicine research.

## 1. Introduction

The Ministry of Health and Welfare of Taiwan estimates that at least 23,000 individuals with spinal cord injury (SCI) reside in Taiwan, and approximately 2500 new spinal cord injuries occur annually 1. The social cost of each patient's lifetime is about TWD 25 million. The most common causes of SCI are traffic accidents, followed by falls, sports accidents and violence. Many animal models, included contusion, compression, dislocation, transection, ischemic, excitotoxic, photochemical and electrolytic models, have been used to study SCI, depending on the aims of the researchers 2. Spinal cord transection is performed after laminectomy to induce complete or targeted interruption of the spinal cord. Complete and incomplete transections result in differences in plasticity in the spinal cord, and incomplete transection reduces the physical harm to the cord and physical separation 3. The marked atrophic response of the affected muscles that is seemingly with incomplete SCI.

SCI causes many pathophysiological changes and has long-term effects on the body 4-6. SCI involves both primary and secondary injury mechanisms. The main mechanism of primary injury is related to the initial mechanical damage. The secondary mechanisms of injury occur after the initial traumatic event and cause tissue destruction during the first few hours after the injury. These secondary mechanisms include ischemia, axonal degeneration, vascular dysfunction, and oxidative stress 7. In addition to the local effects on the spinal cord, SCI also affects the functions of skeletal muscle. Previous studies have reported that a lack of physical activity and the loss of communication between the central nervous system and skeletal muscle due to SCI rapidly reduces skeletal muscle mass 8. SCI patients are at higher risk of developing systemic metabolic disorder, suggesting that the reduction of muscle mass may result in part from systemic metabolic disorder 9, 10. Following SCI, muscle exhibits markedly increased fatigability and altered rates of contraction and relaxation 11. Furthermore, the histopathological changes of muscle also include fiber-type transformation, connective tissues changes and vasculation 8. The status of muscle and locomotion can be assessed to evaluate spinal cord injury and treatment efficacy.

Outbred rodents such as Sprague-Dawley rats and ICR mice are widely used in sport science 12-14. The congenital capacity of animals can be artificially bred to produce stable inherited lines for basic studies with different purposes. Koch et al. divided a founder population into high and low exercise capacity mice by using a treadmill running protocol and further found that the endurance capacity of the high-capacity mice was 1.7 times greater than that of low-capacity mice after 6 generations of breeding 15. In our previous study, we found that the same batch of ICR mice exhibited great variety in exercise performance. By exhaustive swimming exercise test, we divided the mice into high and low congenital exercise ability groups. After 13 generations of breeding, the mice still exhibited differences in exercise ability in the exhaustive swimming exercise test.

Previous studies have showed that animals with a complete spinal cord transection (SCT) can step and stand with practice 16, 17. Activation of the neuromuscular system below the level of the spinal cord lesion by activity-based therapy (ABT) can lead to small improvements in mobility 18. Therefore, the performance of a motor task is improved by practicing a specific task with “challenges” added to a training regimen. However, whether the congenital exercise ability affects spinal cord recovery and histopathological changes of the muscle still remains unclear. In this study, we used selected ICR mice lines of high and low congenital exercise ability to evaluate the effects of congenital exercise ability on the spinal cord and muscle after SCI.

## 2. Material and Methods

### 2.1. Animals

Male outbred ICR mice were purchased from BioLASCO (Control group) (A Charles River Licensee Corp., Yi-Lan, Taiwan). High- and low-congenital exercise ability mice (the HE group and the LE group, respectively) were artificially bred to produce stable inherited lines in the laboratory of Prof. Chi-Chang Huang. For example, the average swimming exhaustion time of the HE group (thirteenth generation) was 97.18 ± 27.97 minutes, while that of the LE group was 2.25 ± 0.30 minutes. All animals were maintained at room temperature (23 ± 2°C) and 55 ± 10% humidity and fed a standard laboratory diet (#. 5001; PMI Nutrition International, Brentwood, MO, USA) with distilled water ad libitum.

**Figure 1:**
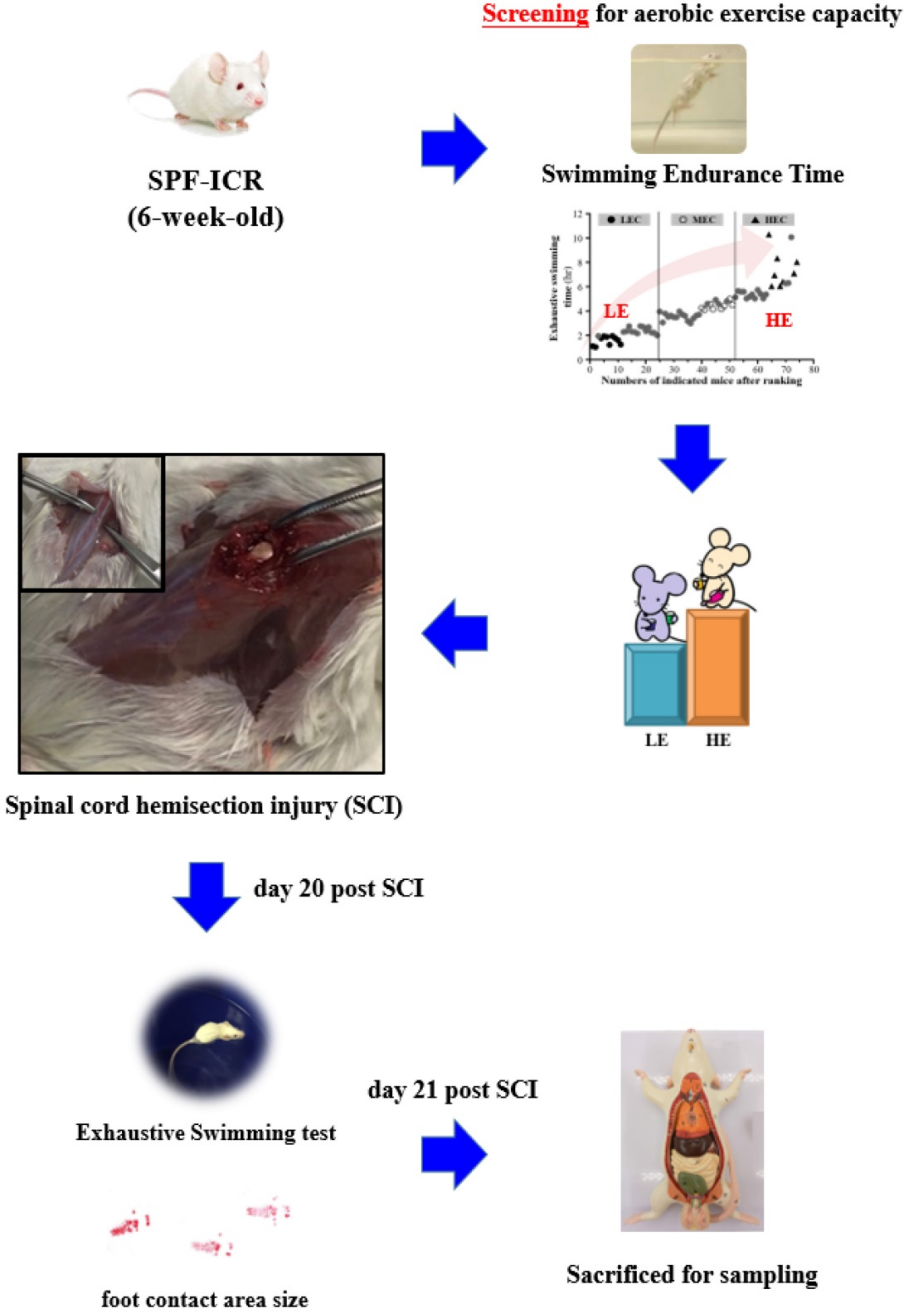
Experimental scheme.

All procedures described in this study were conducted under the approval of the Institutional Animal Care and Use Committee (IACUC) of National Taiwan Sport University after review of the animal study protocols, and the ethics committee of IACUC approved this study (IACUC-10626). All procedures were performed under anesthesia by isoflurane. We made incision along the midline of each 8-week-old male ICR mouse and exposed the dorsal surface of the spinal column from approximately T9 to T10. We performed a laminectomy followed by a left lateral hemi-section with a scalpel and cut the cord, resulting in a moderate injury to the spinal cord. The sham operation consisted of a laminectomy alone. The fascia and skin were sutured closed, and the animals were allowed to recover on a heating pad at 37.8°C. Postoperative treatment consisted of saline (1.0 ml administered subcutaneously) for rehydration. The animals were returned to the preoperative housing conditions, where they began eating and drinking within 3 h of surgery. The mice were assessed with gait analysis and the exhaustive swimming exercise test at day 20 before being euthanasia by 95% CO_2_ asphyxiation at day 21 post SCI. The mice were weighed and the spinal cord and muscle were collected for further analysis. The detailed experimental procedure is illustrated in Fig. 1.

### 2.2. Gait analysis

Gait analysis was performed on a Noldus Catwalk XT (Noldus, Wageningen, The Netherlands). A compliant run met the run criteria: a maximum run duration of 15 s, with a maximum speed variation of 65%. We rejected data in which the animal changed speed or stopped before completing three full gait cycles. Hind limb width and step size were further analyzed.

### 2.3. Exhaustive swimming exercise test

The exhaustive swimming exercise test was performed before and after SCI. A weight equivalent to 5% of body weight in the form of lead fish sinkers was attached to the root of the mouse tail. The tank was maintained at 27°C during the swimming process, and the endurance of each mouse was measured as swimming time, recorded from the beginning of the test to exhaustion, which was defined as uncoordinated movements and failure to return to the surface within 7s. The time of floating, struggling and making necessary movements was assessed until possible drowning and exhaustion.

### 2.4. Histopathological evaluation

Spinal cord and muscle tissue were fixed in 10% neutral buffered formalin for one day, dehydrated, embedded in paraffin, cut into 4μm sections, and stained with hematoxylin and eosin (H&E) for histological examination.

### 2.5. Luxol fast blue staining

Paraffin-embedded sections were deparaffinized and hydrated in water, followed by incubation in Luxol fast blue solution for 24 hours at room temperature. The sections were differentiated by dipping them in lithium carbonate solution and further by alcohol reagent. The sections were incubated in Cresyl Echt Violet for 5 minutes, dehydrated in absolute alcohol, and mounted.

### 2.6. Masson Trichrome staining

Paraffin-embedded sections were deparaffinized and re-fixed in Bouin's solution for 1 hour at 56°C and rinsed with tap water for 5 minutes. The slides were stained in Weigert's iron hematoxylin working solution for 10 minutes, followed by staining in Biebrich scarlet-acid fuchsin solution for 10-15 minutes. The slides were incubated in phosphomolybdic-phosphotungstic acid solution for 10-15 minutes, transferred to aniline blue solution, and stained for 5-10 minutes. The slides were further incubated in 1% acetic acid solution for 2-5 minutes and dehydrated very quickly with 95% ethyl alcohol, absolute ethyl alcohol and clear xylene, after which they were mounted with resinous mounting medium.

### 2.7. Immunohistochemistry staining

The 4-μm formalin-fixed, paraffin-embedded colorectal sections were subjected to deparaffinization, and the slides were submerged in 10 mM citrate buffer (pH 6.0) until boiling for antigen retrieval. Hydration was carried out prior to quenching of endogenous peroxidase activity (3% H_2_O_2_ in methanol for 10 min). The sections were incubated overnight with the primary anti-glial fibrillary acidic protein (#ab7260, Abcam, MA, United States) diluted at 1:200. The slides were subsequently treated with Picture^TM^ HRP Polymer conjugate at room temperature for 20 min. HRP localization was visualized using a Liquid DAB+ Substrate Chromogen System (#K3468, Dako, CA, United States).

### 2.8. Statistical analysis

All data are presented as the mean ± standard deviation (SD). GraphPad Prism 6 version 6.01 for Windows (La Jolla, CA, USA) was used to analyze differences between groups. One-way analysis of variance (ANOVA) was used to compare multiple groups, followed by Fisher's LSD. Statistical significance was set at p < 0.05.

## 3. Results

### 3.1. The HE group and LE group exhibited differences in congenital exercise ability as compared with the control group

**Figure 2:**
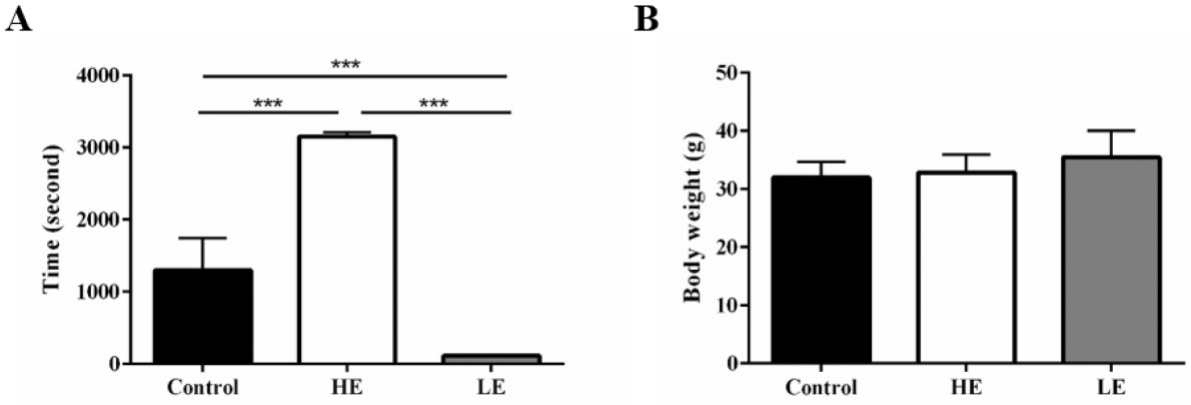
** Stable inherited lines of mice exhibited differences in congenital exercise ability.** (A) exhaustive swimming time and (B) terminal body weight of the control, HE and LE group. Data are expressed as the mean ± SD and were analyzed by two-way ANOVA followed by Turkey's post hoc test. *p < 0.05, n = 7.

To confirm the phenotype of the congenital exercise ability, all mice performed the exhaustive swimming exercise test with 5% body-weight loading before SCI. The average exhaustive swimming time of the HE group was 3152 ± 60.5 seconds, while that of the LE group was only 114 ± 3.4 seconds. For comparison, the average exhaustive swimming time of the control group was 1301 ± 443.4 seconds (Fig. 2A). Additionally, body weight was not significantly different among these three groups (Fig. 2B). The results indicated that the congenital exercise ability could be artificially bred to produce stable inherited lines.

### 3.2. HE group still exhibited higher exercise ability than the LE group after SCI

**Figure 3:**
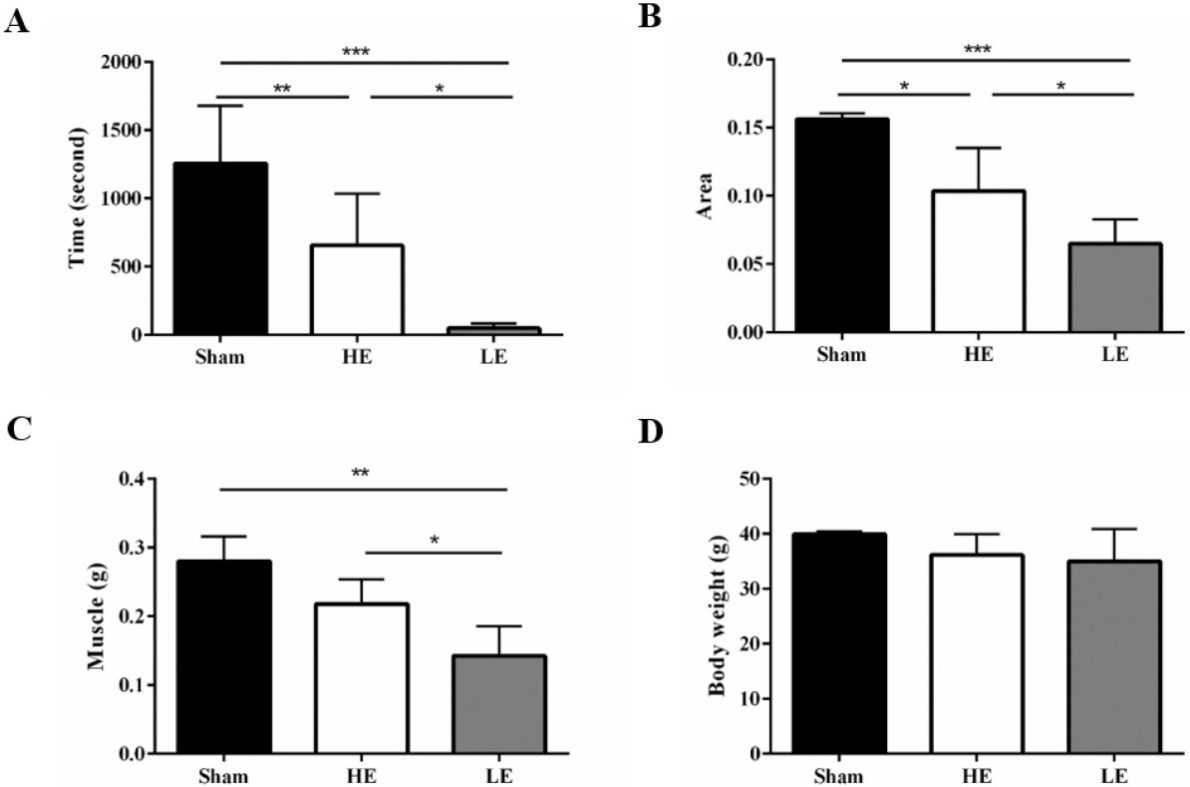
** HE group still exhibited higher exercise ability compared with the LE group after SCI.** (A) exhaustive swimming time (B) foot contact area size by gait analysis. (C) Muscle weight and (D) terminal body weight of the control, HE and LE group. Data are expressed as the mean ± SD and were analyzed by two-way ANOVA followed by Turkey's post hoc test. *p < 0.05, n = 5.

At day 20 post SCI, all mice performed the exhaustive swimming exercise test with 5% body-weight loading. Both the HE group and the LE group exhibited decreased exhaustive swimming times compared with the Sham group, which indicated that the SCI model was successfully established in this study. Interestingly, the exhaustive swimming time of the HE group was about 13.3 folds higher than that of the LE group (Fig. 3A). Similar results in foot contact area size were also observed in gait analysis. The area was larger in the HE group than in the LE group (Fig. 3B). The muscle weights of the HE and Sham groups were greater than that of the LE group; however, there was no difference between those of the HE and Sham groups (Fig. 3C). There were no differences the terminal body weights of the three groups (Fig. 3D). These results indicated that after SCI, the HE group still exhibited a higher exercise ability than that of the LE group.

### 3.3. Congenital exercise ability may not affect recovery of the spinal cord

**Figure 4:**
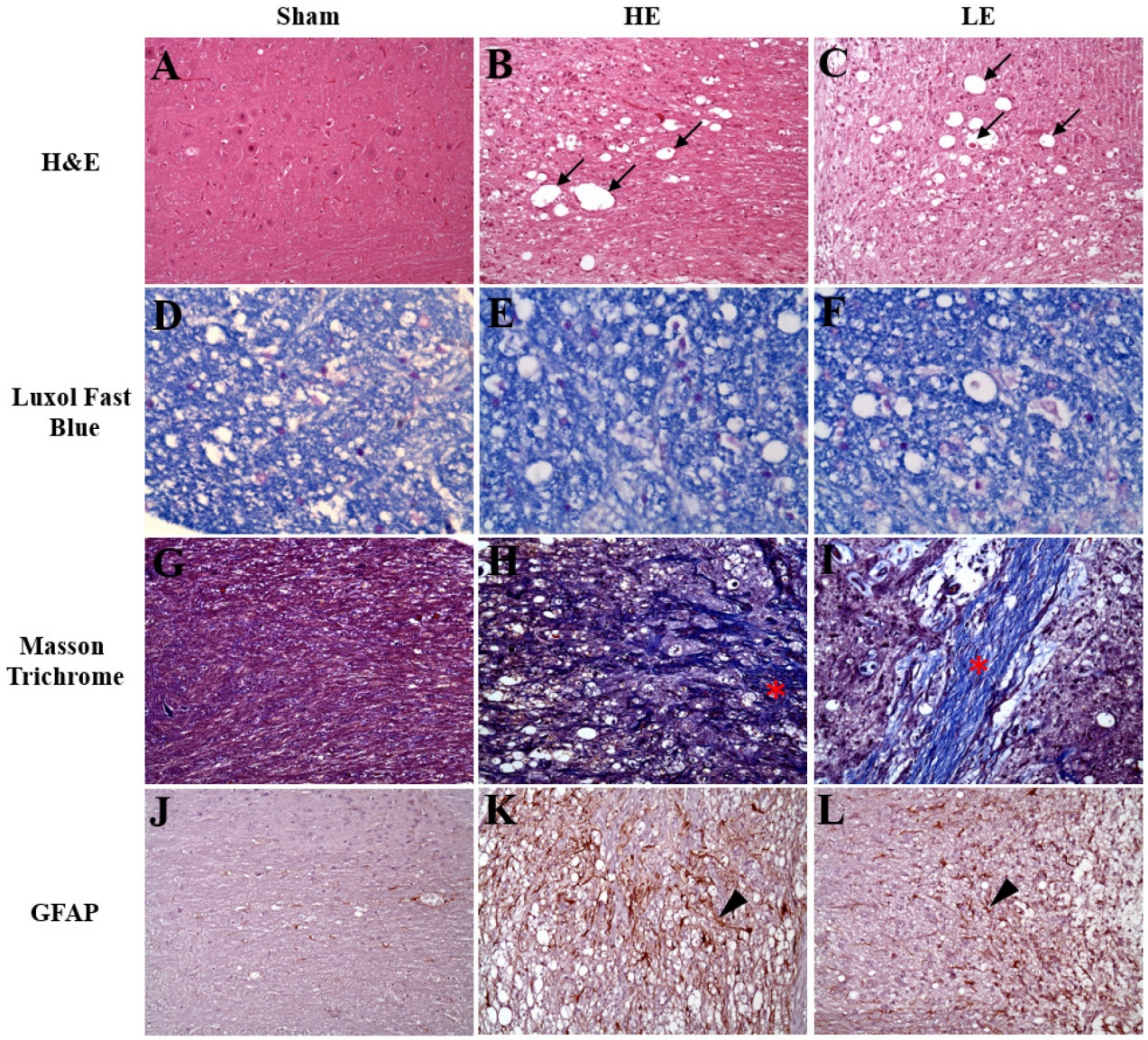
** Histopathology and immunohistochemistry staining of spinal cord.** Sham (A, D, G, J), HE (B, E, H, K) and LE (C, F, I, L) groups. (A-C) The neuro-fiber vacuolation (black arrow) was observed in the HE and LE groups. (D-F) The demyelination was found in the HE and LE groups. (G-I) The fibrous scars (asterisk) was found in the HE and LE groups. (J-L) The GFAP-positive astrocytes (arrow head) was found in the HE and LE groups. magnification × 200.

As indicated in Fig. 4, the results of H&E staining revealed neuro-fiber vacuolation and myelin degeneration of the spinal cord in the HE and LE groups, but not in the Sham group. Additionally, more spheroids were observed in the HE and LE groups than in the Sham group. Further analysis by Luxol fast blue staining also confirmed myelin degeneration in the HE and LE groups. Masson trichrome staining showed more fibrous scars in the surgical sites of the HE and LE groups than in those of the Sham group. However, no differences in histopathological changes were found between the HE and LE groups. Although assessment of neuron regeneration by immunohistochemistry staining of GFAP showed positive signals in the HE and LE groups. However, there were no differences between the HE and LE groups. These results revealed that congenital exercise ability may not affect recovery of the spinal cord.

### 3.4. HE group exhibited less severity of muscle atrophy than did the LE group after SCI

**Figure 5:**
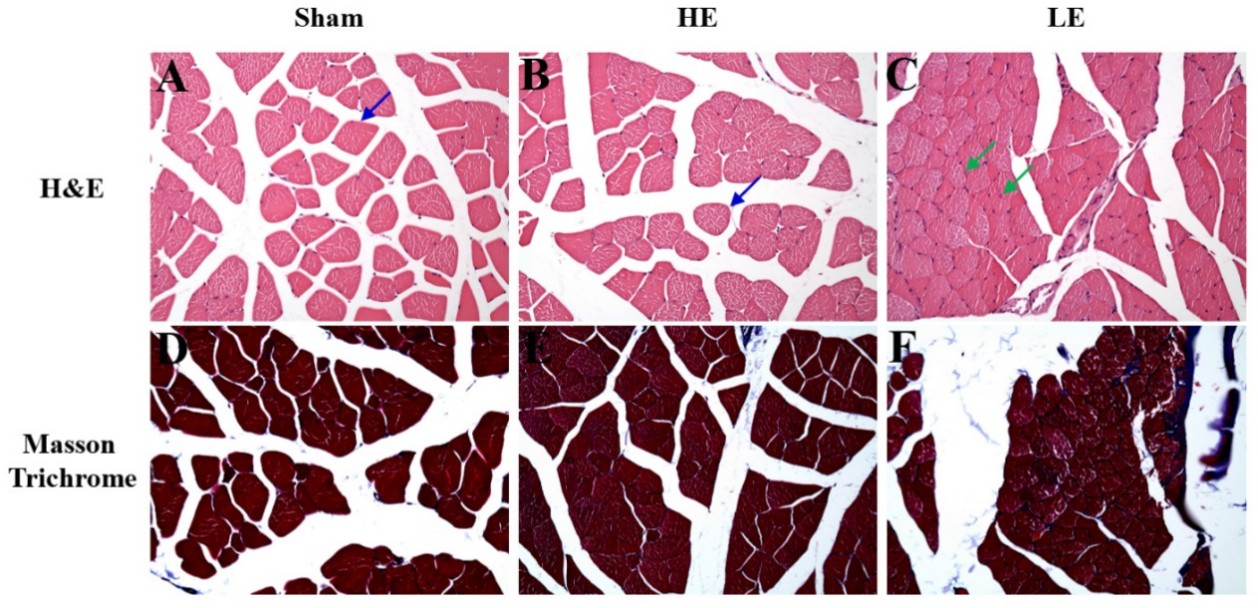
** Histopathology of muscle by H&E staining and Masson Trichrome staining.** (A-C) The normal structure of muscle fiber (blue arrow) was observed in the Sham and HE groups. However, the slight to moderate of muscle atrophy (green arrow) was appeared in the LE group. (D-F) The more fibrous tissue was found in the LE group than in SHAM and HE groups. magnification × 200.

To assess the effects of congenital exercise ability on muscle after SCI, we observed the histopathological changes by H&E and Masson Trichrome staining. As shown in Fig. 5, few atrophy patterns with angulated atrophic fiber were observed in the HE and Sham groups. However, obvious angulated atrophic fiber was observed in the LE group. Masson Trichrome staining revealed that the HE group had little fibrous connective tissue among the muscle fibers. In contrast, obvious fibrous connective tissue was observed in the LE group. No fibrous connective tissue was observed in the Sham group. These results indicated that congenital exercise ability may affect the histopathological changes in muscle after SCI.

## 4. Discussion

In the present study, we bred differences in congenital exercise ability from founder ICR mice selected by exhaustive swimming exercise test. Even after SCI, performance on gait analysis and the exhaustive swimming exercise test was better in the HE group than in the LE group. However, no studies have assessed the effects of congenital exercise ability on spinal cord injury. The results are divided into two parts, namely, spinal cord and muscle, for discussion.

Previous studies have shown that SCI can cause nerve damage and systemic inflammation. Numerous studies have also shown that exercise can help SCI patients to recover 19-21. Yan Sun et al. indicated that exercise can ameliorate SCI damage through NF-κB signaling pathways 22. Several studies have also indicated that inhibition of inflammation can improve axonal regeneration 23, 24. In the present study, as the evidence is not clear yet in regards to the direct relationship between congenital exercise ability and inflammation, fibrosis, or demyelination. Conversely, exercise intervention after SCI may play an important role in spinal cord recovery.

In the present study, we found that after SCI, the muscle weight of the HE group was greater than that of the LE group. In our previous study, we found no differences in muscle weight between the HE and LE groups without SCI 25. These results indicated that congenital exercise ability may influence the pathology of SCI in muscle tissue. Indeed, our H&E and Masson Trichrome staining results indicated reduced muscle atrophy and fibrosis of the HE group as compared with those of the LE group. A previous study has reported that increasing the uptake of glucose and fatty acids by muscle cells and enhancing the metabolism of glucose and lipids can increase the skeletal muscle mass and strength and enhance the endurance of the body 26. In our previous study, high exercise capacity mice exhibited greater glucose tolerance and maintained better homeostasis than low and medium exercise capacity mice, indicating better glucose utilization in the high exercise capacity mice 25. Based on this evidence and the fact that the mice were bred from the same founder mice, better glucose utilization may contribute to amelioration of muscle atrophy.

Based on the above results, in this experiment, a mouse model of spinal cord injury has been developed to demonstrate that differences in congenital exercise capacity ameliorate muscle atrophy but not the recovery of spinal cord injury. We suggest that congenital exercise capacity may not affect the regeneration of the nerve cells of the spinal cord or the reduction of inflammation. Surprisingly, the major effects were the reduction of muscle injury, including muscle atrophy and fibrosis, in the HE group as compared with the LE group after SCI. In conclusion, our results point out the effects of congenital exercise ability on spinal cord injury. This study may have an impact on understanding the interference of congenital features.
